# Global Navigation Satellite System Multipath Mitigation Using a Wave-Absorbing Shield

**DOI:** 10.3390/s16081332

**Published:** 2016-08-22

**Authors:** Haiyan Yang, Xuhai Yang, Baoqi Sun, Hang Su

**Affiliations:** 1 National Time Service Center, Chinese Academy of Sciences, Xi’an 710600, China; yyang@ntsc.ac.cn (X.Y.); sunbaoqi@ntsc.ac.cn (B.S.); suxing11@mails.ucas.ac.cn (H.S.); 2 University of Chinese Academy of Sciences, Beijing 100049, China; 3 Key Laboratory of Precision Navigation and Timing Technology, National Time Service Center, Chinese Academy of Sciences, Xi’an 710600, China

**Keywords:** global navigation satellite system (GNSS), wave-absorbing shield, multipath, single-point positioning (SPP)

## Abstract

Code multipath is an unmanaged error source in precise global navigation satellite system (GNSS) observation processing that limits GNSS positioning accuracy. A new technique for mitigating multipath by installing a wave-absorbing shield is presented in this paper. The wave-absorbing shield was designed according to a GNSS requirement of received signals and collected measurements to achieve good performance. The wave-absorbing shield was installed at the KUN1 and SHA1 sites of the international GNSS Monitoring and Assessment System (iGMAS). Code and carrier phase measurements of three constellations were collected on the dates of the respective installations plus and minus one week. Experiments were performed in which the multipath of the measurements obtained at different elevations was mitigated to different extents after applying the wave-absorbing shield. The results of an analysis and comparison show that the multipath was mitigated by approximately 17%–36% on all available frequencies of BeiDou Navigation Satellite System (BDS), Global Positioning System (GPS), and Global Navigation Satellite System (GLONASS) satellites. The three-dimensional accuracies of BDS, GPS, and GLONASS single-point positioning (SPP) were, respectively, improved by 1.07, 0.63 and 0.49 m for the KUN1 site, and by 0.72, 0.79 and 0.73 m for the SHA1 site. Results indicate that the multipath of the original observations was mitigated by using the wave-absorbing shield.

## 1. Introduction

Multipath effects are a key parameter in global navigation satellite system (GNSS) observation quality. These effects have different impacts on different frequencies. It is difficult to eliminate these impacts by differential technology or establishing a mathematical model [1,2,3,4,5,6]. Multipath effects are a major error source that jeopardizes GNSS positioning accuracy. The effects influence the accuracy of single-point positioning (SPP); moreover, severe multipath effects slow the convergence of precision point positioning (PPP) [7,8]. Therefore, mitigating the multipath influence is critical for improving GNSS positioning accuracy.

Many methods relating to GNSS multipath and its mitigation have been proposed and developed. These methods can be respectively classified as antenna, signal, and measurement processing [7,8,9,10,11,12,13,14,15]. Improvement of antenna design mainly focuses on the lower gain at low satellite elevations, such as choke-ring antennas and ground planes. To model multipath variation, the measurement processing involved in the above studies leverages the relationship between the multipath and elevation, and between the multipath and signal-to-noise ratio (SNR) measurements. Other methods, such as wavelet decomposition and sidereal filtering, have also been used to denoise and mitigate multipath effects [10,11].

The above research has focused on hardware design and measurements post-processing methods. However, it should be noted that careful site selection and improved observation conditions are the best remedies for multipath effects. We therefore propose installation of a wave-absorbing shield with a diameter of 1.2 m around the antennas of GNSS sites to improve the local observation conditions and there by mitigate the multipath effects. Absorbing material is typically applied to the antenna itself to mitigate multipath, while some antennas on the market include absorbing material to attenuate the surface waves [12,14,15].

This paper is organized as follows: the structure and main design parameters of the proposed wave-absorbing shield are first introduced in greater detail. In Section 3 and Section 4, we describe the multipath combination of code and carrier phase observables, as well as the multiple perspective analysis that was conducted on the time series of multipath observations of BeiDou Satellite Navigation System (BDS), Global Positioning System (GPS), and Global Navigation Satellite System (GLONASS) satellites over the period of two weeks. To verify the proposed method, SPP was conducted. The positioning results before and after multipath mitigation are compared in Section 5. In Section 6, our conclusions are presented.

## 2. Wave-Absorbing Shield

**Figure 1:**
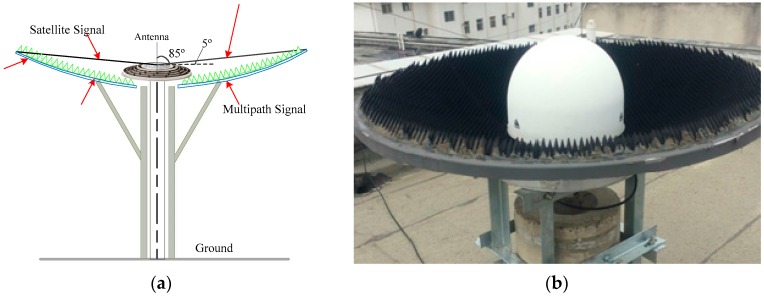
Structure (**a**) and photo (**b**) of the wave-absorbing shield (in Figure 1a, green represents the wave-absorbing material, blue represents the wave-absorbing isolation plate, gray represents the support structure, the circular shape represents the choke-ring antenna).

The proposed shield is primarily composed of a wave-absorbing isolation plate and wave-absorbing materials. As shown in Figure 1, a 1 mm thick aluminum wave-absorbing isolation plate is attached to a 1.2 m diameter parabolic metal plate with a 5° angle between the edge of the parabolic metal plate and the phase center of the GNSS antenna. The surface of the wave-absorbing isolation plate is covered with wave-absorbing material. In the design of wave-absorbing shield, performance of multipath mitigation must be considered without effecting GNSS observations collected. The installation angle was set as 5° according to the cutoff angle, which is typically set **as** about 5°–15° during GNSS data collecting and processing, and guarantees the integrity of collected observations. The diameter of the wave-absorbing shield is the result of many factors, such as mitigation performance, performance and price rate, easy to install, and so on.

Direct and reflected signals with an elevation angle of less than 5° (for the zenith distance of larger than 85°) are isolated by the wave-absorbing isolation plate, which requires excellent isolation performance. Isolation performance refers to the total attenuation value of the radial electromagnetic radiation energy after passing through a barrier. This value is expressed in dB. The formula for calculating the isolation performance is [16,17]:

(1) SE=R+A+K

where *SE* is the isolation performance, *R* is the reflection loss on both sides of the wave-absorbing isolation plate, and *A* is the absorption loss of the isolation plate. In addition, *K* is the internal wave emission correction factor of the isolation plate. Among these parameters, *A* is the most important for the isolation performance; when *A* > 10 dB, the *R* and *K* values are usually ignored. According to Schelkunoff's theory of electromagnetic shielding and shielding efficiency [17,18], the formula for *A* is $A=1.314\text{t}\sqrt{f\sigma_{x}\mu_{x}}$, where, *t* denotes the thickness (cm) of the isolation plate, *f* is the frequency (Hz), $\sigma_{x}$ is the relative conductivity of the absorbing isolation plate material, and $\mu_{x}$ is the relative permeability of the wave-absorbing isolation plate material.

Table 1 outlines the test results for the isolation performance of a 1 mm thick aluminum wave-absorbing isolation plate on different frequencies. According to Equation (1), the isolation performance of the wave-absorbing shield is more than 120 dB in 1~3 GHz frequency range, which is excellent. Therefore, the wave-absorbing shield can efficiently shield disturb signals from low elevation.

Wave-absorbing materials mainly absorb signals that are reflected by the wave-absorbing isolation plate and direct signals from satellites and other objects. Ten-centimeter-thick cone rubber absorbing materials were used in this application out of the consideration for the environmental adaptability. Test results show that the absorption performance for vertical incident waves is superior to 15 dB in the 1~3 GHz frequency range. The used materials match the electromagnetic wave frequency characteristics of the GNSS satellites’ transmission signals. Therefore, GNSS signals are largely transmitted from the surface to the interior of the material, which reduces signal reflection and absorbs or attenuates the internal incident disturb signals [19,20].

Compared with the methods in literature [12], the advantages of our methodology are as follows. Firstly, the shape of the device adopts a ground plane instead of circular paraboloid. Secondly, using rubber-absorbing materials rather than foam ensures normal work outside. Thirdly, the present method synthetically utilizes the circular paraboloid and rubber absorbing materials together, and not isolation.

## 3. Measurement Collection and Validity

**Figure 2:**
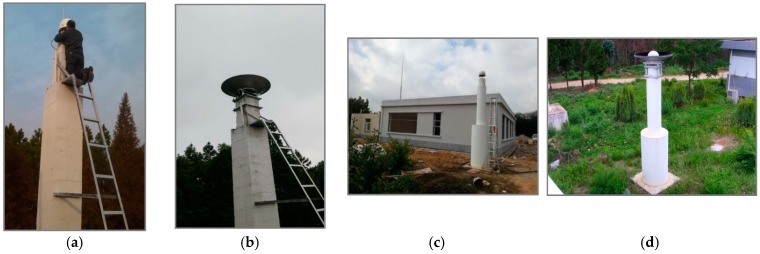
SHA1 site: without (**a**) and with (**b**) the wave-absorbing shield; KUN1 site: without (**c**) and with (**d**) the wave-absorbing shield.

The experiments were performed at the international GNSS Monitoring and Assessment System (iGMAS) [21] sites: SHA1 (Shanghai, China, 31°5.98′ N, 121°12.03′ E) and KUN1 (Kunming, China, 25°1.80′ N, 102°47.47′ E). The sites were equipped with Unicore UB4B0I multi-frequency multi-GNSS receivers and NOV750 choke-ring antennas. The antennas were mounted on the top of a concrete pillar and were located higher than other adjacent buildings. However, the tall trees, which were planted at a distance of at least 30 m from the antennas, were a main source of reflected signals (see Figure 2). Based on the test, the multipath effect at the two sites was relatively low.

The wave-absorbing shields were installed on 28 April 2016 and 8 May 2016, at the respective SHA1 and KUN1 sites. Figure 2 shows the installed wave-absorbing shield and the Unicore multi-GNSS multi-frequency receivers and antennas that were used in the experiment. According to the design requirements, signals below the satellite elevation of 5° will be lost owing to obscuration by the wave-absorbing shield.

Measurements for the present study were collected from the two sites on the triple-frequency GPS (L1, L2, L5) and BDS (B1, B2, B3), as well as the dual-frequency GLONASS (G1, G2). The measurements were collected on the dates of the respective wave-absorbing shield installations plus and minus one week at a sampling interval of 30 s.

**Figure 3:**
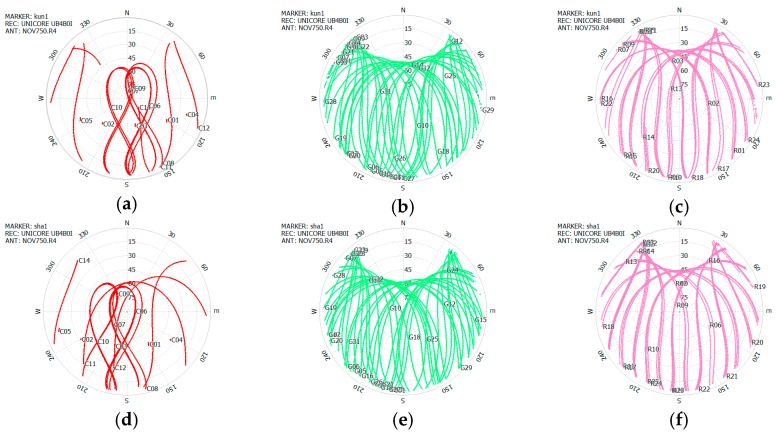
Sky plot of three constellations after the wave-absorbing shield installation at the KUN1 site on 11 May 2016, and SHA1 site (down) on 4 May 2016. KUN1: BDS (**a**); GPS (**b**) and GLONASS (**c**); SHA1: BDS (**d**); GPS (**e**) and GLONASS (**f**). The cutoff elevation is 5°.

Figure 3 shows the sky plots of BDS, GPS, and GLONASS at the KUN1 and SHA1 sites after the wave-absorbing shield installation. The cutoff elevation was set as 5°. From these figures, we can see the elevation and azimuth of each satellite observed at KUN1 and SHA1 site. The signals in all directions can be consecutively tracked above 5° elevation. It should be noted that the wave-absorbing shield has no influence on the measurements collected. In addition, the tracking signals of the GPS, GLONASS, and BDS (C11–C14) medium earth orbit (MEO) satellites are lost when they move below an elevation of 5°. BDS geostationary orbit (GEO) satellites (C01–C05) always remain over the equator. BDS inclined geosynchronous orbit (IGSO) satellites (C06–C10) have a ground track of figure-eight loops with a mean longitude. A data gap exists on account of the satellite elevation measuring below 5°.

**Figure 4:**
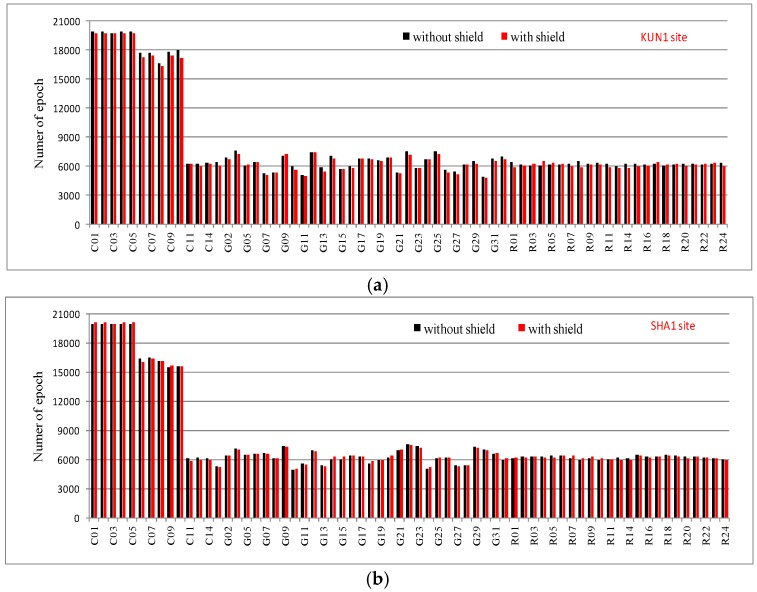
The number of valid observations from KUN1 (**a**) and SHA1 (**b**) sites for all satellites before and after the wave-absorbing shield installation (the length of each series is 7 days, interval is 30 s).

Figure 4 shows the validity of BDS, GPS, and GLONASS observations collected from KUN1 and SHA1 sites. The length of each dataset is seven days. The bars represent total numbers of epochs that dual/triple-frequency code and carrier phase observations were actually processed in paper. Through the comparison of the total numbers of epochs in two terms for each station, it was determined that there is not much difference. Furthermore, according to statistics, the observations’ validity rates for all satellites are more than 95%.

## 4. Multipath Observations and Analysis

The multipath combination is often used to assess code multipath and noise level of a receiver [3,6,9]. The maximum influence of the multipath effect on the carrier phase is typically a quarter of the wavelength [17], which is two orders of magnitude smaller than its influence on the code. In the case of single site—without considering the multipath effects of the carrier phase—the code multipath can be estimated with a linear combination of code and carrier phase [7,22,23]:

(2) $$mp_{i}=P_{i}-\Phi_{i}+2\lambda_{i}^{2}\frac{\Phi_{j}-\Phi_{i}}{\lambda_{j}^{2}-\lambda_{i}^{2}}+\varepsilon_{i}$$

where *mp* is the multipath effect of the code in meters, *P* is the measurement of code in meters, Φ is the measurement of carrier phase in meters, and *λ* is the respective wavelengths in meters. In addition, ε is the measurement noise and *j* is a frequency different from *i*.

In the above combination, the code multipath cannot be effectively separated from the measurement noise, including the influences of carrier phase ambiguity and equipment hardware delay. For GNSS receivers, the deviation range of the hardware delay is slightly over observation periods; thus, monthly averages were used for corrections in precise positioning. The ambiguity is a constant in the observation period, provided that no cycle slip occurs.

Under the above conditions, the carrier phase can be used as a highly precise code to eliminate system deviations. The assessment of code multipath can be represented as:

(3) $$MP_{i}=mp_{i}-mp_{iT}$$

where $mp_{iT}$ is the mean value of $mp_{i}$ during time *T*. The influence of the constant term is eliminated by subtracting the mean term. Therefore, $MP_{i}$ actually reflects the statistical characteristics of code multipath rather than the absolute values.

**Figure 5:**
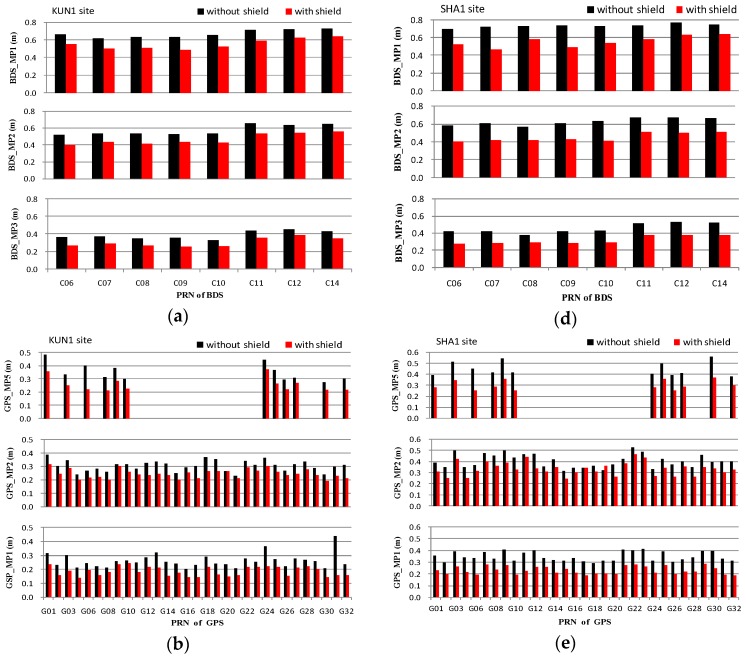

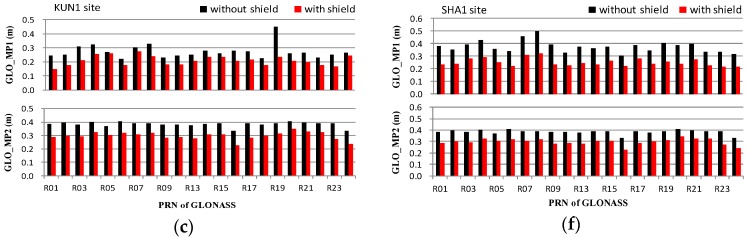
Standard deviations of the code multipath for all available satellites and comparison of states before and after mitigation. KUN1: BDS (**a**), GPS (**b**) and GLONASS (**c**); SHA1: BDS (**d**), GPS (**e**) and GLONASS (**f**).

The statistics of the code multipath on all frequencies created for all available satellites are shown in Figure 5. These include BDS MEO (C11–C14) and IGSO (C06–C10), GPS, and GLONASS satellites. It can be seen from the figure that the multipath of all satellites are mitigated after the wave-absorbing shield installation, however, a difference exists among different satellites, which is primarily due to the different elevations of the satellites. The standard deviations of multipaths are about 0.25–0.73 m, 0.14–0.45 m, and 0.14–0.46 m for BDS, GPS and GLONASS, respectively. The averages of the standard deviations on available frequencies of each constellation are listed in Table 2. The average values of multipath mitigation using the wave-absorbing shield are about 0.08–0.17 m, 0.05–0.16 m, and 0.08–0.14 m for BDS, GPS and GLONASS, respectively, the corresponding mitigation ratios are about 17.5%–28.9%, 16.7%–35.1% and 24.5%–35.9%, respectively.

**Figure 6:**
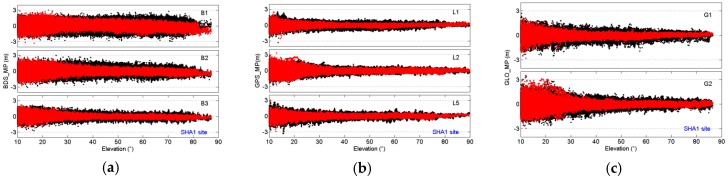
Comparison of code multipath against elevation angles for BDS IGSO and MEO (**a**); GPS (**b**); and GLONASS (**c**) at SHA1 site. Red and black represent before and after mitigation, respectively.

Figure 6 shows the multipath variations for BDS IGSO and MEO, GPS, and GLONASS with their corresponding elevation angles at the SHA1 site. The variation trends of the multipath before and after the wave-absorbing shield installation are the same: the multipaths decrease with an increasing elevation angle. However, the distributions of multipaths show a significant difference after the wave-absorbing shield installation.

**Figure 7:**
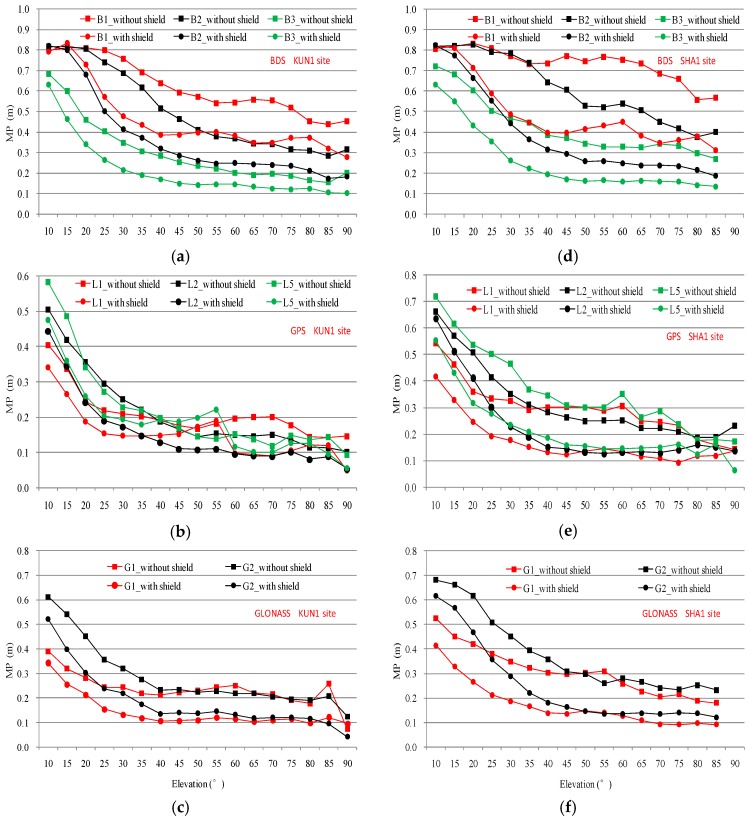
Standard deviation of the code multipath against elevation for three constellations on all available frequencies and comparison of states before and after mitigation. KUN1: BDS (**a**); GPS (**b**) and GLONASS (**c**); SHA1: BDS (**d**); GPS (**e**) and GLONASS (**f**).

Figure 7 shows the variation of multipath with elevation for BDS, GPS, and GLONASS measurements at KUN1 and SHA1 sites. The same color lines represent the same frequency, and the square and circular logos respectively represent the states before and after the wave-absorbing shield installation. Multipath was calculated for each 5° increment in the satellite elevation angles. For example, the multipath at an elevation angle of 50° represents a statistical value of the multipath between the satellite elevation angles from 47.5° and 52.5°. We can easily compare the multipath between the states before and after mitigation at a given elevation angle according to the results shown in Figure 7. As we can see from Figure 7, except BDS B1 and B2 at the elevation below 20°, a significant multipath mitigation exists on all available frequencies.

**Figure 8:**
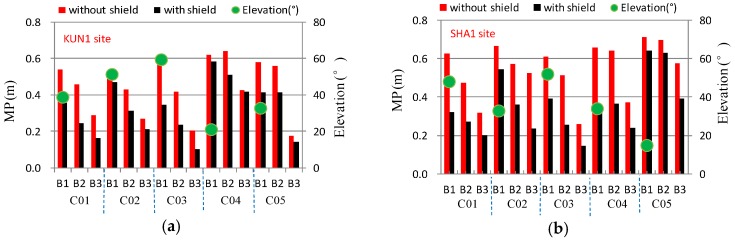
Multipath on all frequencies and elevations for BDS GEO satellites, and comparison of states before and after mitigation at KUN1 (**a**) and SHA1 (**b**).

BDS GEO satellites are almost stationary relative to the tracking sites on the earth. They can be continuously observed for the two sites. The multipath effect on the accuracy of GEO measurements with low elevations is much more significant than those of IGSO and MEO [9,10,11]. The standard deviations of code multipath on all frequencies for BDS GEO satellites are shown in Figure 8. It shows that the mean mitigation rate of multipath was about 30%, but the multipath mitigation and elevation for each satellite were obviously different. For example, the elevations of GEO satellites C03 and C04 are approximately 59.5° and 21.0° observed at KUN1, respectively, corresponding to a mitigation ratio of approximately 45.0% and 9.5%. The elevations of GEO satellites C03 and C05 are approximately 52° and 15° observed at SHA1, respectively, corresponding to a mitigation ratio of approximately 43.0% and 16.9%.

**Figure 9:**
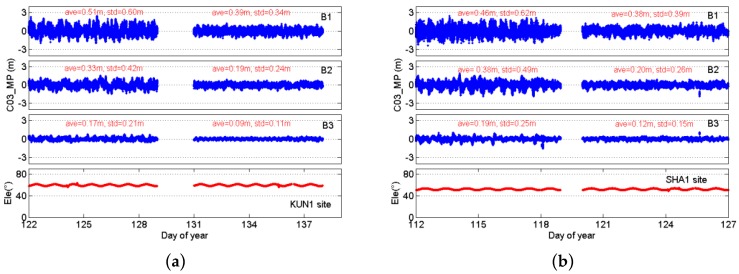
Multipaths of B1, B2, and B3 code and elevations for the BDS GEO satellites C03 at KNU1 (**a**) and SHA1 (**b**) sites, and comparison of states before and after mitigation. The data gaps represent the date of the wave-absorbing shield installation.

Figure 9 presents the time series of B1, B2, and B3 multipath for a typical GEO satellite (BDS C03), and provides the absolute averages and standard deviations of multipath before and after the wave-absorbing shield installation. The sequence of multipath for GEO satellites is always continuous. By comparison, the dispersions of multipath are significantly reduced using the wave-absorbing shield.

**Figure 10:**
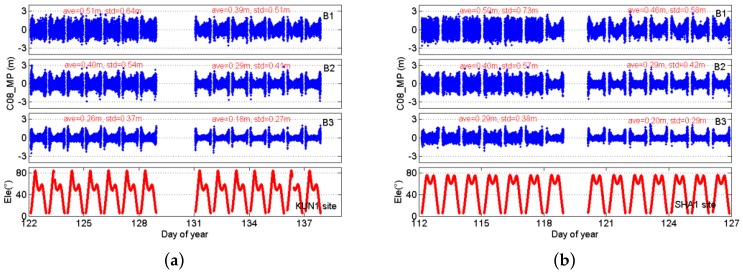

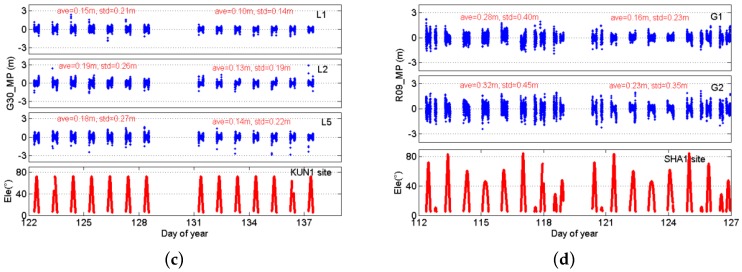
Multipaths of B1, B2, and B3 code and elevations for KUN1: IGSO C08 (**a**) and SHA1 IGSO C08 (**b**); MEO G30 (**c**) and MEO R09 (**d**); and comparison of states before and after mitigation. The data gaps represent the date of the wave-absorbing shield installation.

Figure 10 depicts the time series of B1, B2, and B3 multipath for a typical IGSO satellite (BDS C08) and two MEO satellites (GPS G30 and GLONASS R09) observed at KUN1 and SHA1 sites, and provides the statistics of multipath before and after the wave-absorbing shield installation. The statistics show the absolute averages and standard deviations of multipaths are smaller after mitigation than before mitigation. Based on these figures, a striking contrast is apparent between the states before and after the wave-absorbing shield installation. The multipath mitigation of these satellites using the wave-absorbing shield is not observable at both ends of each observation arc compared to that at the middle of each observation arc.

## 5. Single-Point Positioning Results

To assess the performance of the wave-absorbing shield, GPS, GLONASS, and BDS SPP solutions were computed based on the collected datasets from the KUN1 and SHA1 sites. Satellite orbit, clock error corrections, and ionosphere delay were calculated using the corresponding broadcast ephemeris. Tropospheric corrections were considered by using the Saastamoinen model [24] with standard meteorological values. Receiver clock parameters and site coordinates were estimated for each observation epoch with respect to BDS, GPS, and GLONASS. The positioning results were converted to the site-centric coordinate system and compared with the GPS PPP (accuracy of better than 10 cm) to assess the positioning error.

**Figure 11:**
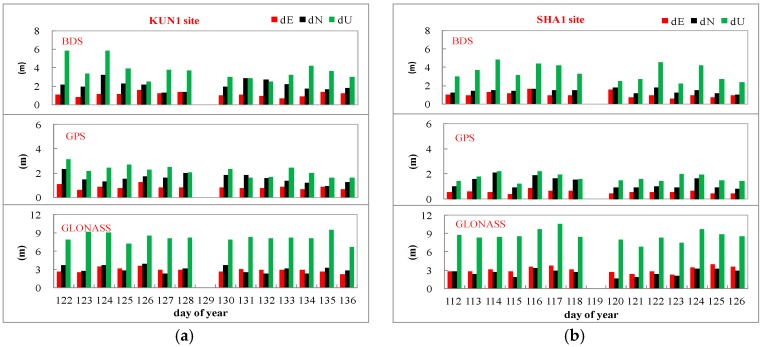
Standard deviations of BDS, GPS and GLONASS SPP solution of KUN1 (**a**) and SHA1 (**b**) with respect to GPS precision point positioning (PPP) coordinates. Day 129 and day 119 are the dates of the wave-absorbing shield installations for KUN1and SHA1 sites, respectively.

The standard deviations of SPP solution for BDS, GPS, and GLONASS with respect to GPS PPP coordinates are showed in Figure 11. Difference of the standard deviation of SPP before and after multipath mitigation, including north, east, and up components, is shown below.

- BDS: north, east, up, and three-dimensional
  ○ SHA1: 15, 31, 64 cm, and 0.72 m
  ○ KUN1: 7, 15, 106 cm, and 1.07 m
- GPS: north, east, up, and three-dimensional
  ○ SHA1: 62, 11, 48 cm, and 0.79 m
  ○ KUN1: 27, 16, 56 cm, and 0.63 m
- GLONASS: north, east, up, and three-dimensional
  ○ SHA1: 19, 33, 63 cm, and 0.73 m
  ○ KUN1. 33, 28, 23cm, and 0.49 m

Through the comparison of statistics obtained before and after mitigation, it is demonstrated that a higher accuracy of SPP can be realized using the multipath mitigation measurements. The wave-absorbing shield installation is therefore attractive for applications in continuously operating reference sites.

## 6. Conclusions

In this paper, we proposed a method to mitigate code multipath by installing a wave-absorbing shield around the antennas of GNSS tracking sites. BDS, GPS, and GLONASS data from iGMAS KUN1 and SHA1 sites observed during the respective dates of the wave-absorbing shield installation, plus and minus one week, were used to examine the utility of the proposed method.

By comparing and analyzing the experimental results, the feasibility and validity of the multipath mitigation method were proved. The multipath linear combination was calculated and used to assess the mitigation values of the code multipath. In the results, the mitigation ratios on all available frequencies of three different constellations are close to an approximate varying range from 17% to 36%. Compared with the mitigation ratio of 7%–24% in the literature [12], the present method has better results. Also, there was a significant multipath mitigation at different elevation ranges for most frequencies.

The results of the BDS, GPS, and GLONASS SPP experiment demonstrate that the positioning precision is improved by the multipath mitigation measurements. After applying the wave-absorbing shield, the three-dimensional accuracy of BDS, GPS, and GLONASS solutions were respectively improved by 1.07, 0.63, and 0.49 m for KUN1 site, and by 0.72, 0.79 and 0.73 m for SHA1 site. It should be noted that the statistical values in the present study relate to the receivers and antennas used in the experiments and different results may be produced with other types of receivers and antennas.

We herein focused on the multipath mitigation of code measurements. The carrier phase multipath may be mitigated in a similar way. This subject will be investigated in our subsequent research. Meanwhile, we contend that the presently proposed wave-absorbing shield can be deployed as a useful approach for mitigating multipath of measurements from continuously operating GNSS tracking sites.

## Figures and Tables

**Table 1:** Performance of the wave-absorbing isolation plate.

Frequency (Hz)	$\sigma_{x}$	$\mu_{x}$	A (dB)	SE (dB)
1.0 × 10^9^	0.17	1	1713.6	>120
1.5 × 10^9^	0.17	1	2098.8	>120
2.0 × 10^9^	0.17	1	2423.5	>120
2.5 × 10^9^	0.17	1	2708.9	>120
3.0 × 10^9^	0.17	1	2968.1	>120

**Table 2:** Standard deviations (m) of the code multipath for BDS IGSO and MEO, GPS, and GLONASS at KUN1 and SHA1 sites and comparison of states before and after mitigation.

Site	BDS			GPS			GLONASS		Note
	B1	B2	B3	L1	L2	L5	G1	G2	
SHA1	0.73	0.62	0.45	0.37	0.42	0.47	0.39	0.49	without shield
	0.56	0.45	0.32	0.24	0.35	0.31	0.25	0.37	with shield
	23.3%	27.4%	28.9%	35.1%	16.7%	34.0%	35.9%	24.5%	ratio
KUN1	0.67	0.57	0.39	0.26	0.31	0.35	0.30	0.40	without shield
	0.55	0.47	0.31	0.21	0.25	0.29	0.22	0.30	with shield
	17.9%	17.5%	20.5%	19.2%	19.4%	17.1%	26.7%	25.0%	ratio
